# Determination of Therapeutic Equivalence of Generic Products of Gentamicin in the Neutropenic Mouse Thigh Infection Model

**DOI:** 10.1371/journal.pone.0010744

**Published:** 2010-05-20

**Authors:** Andres F. Zuluaga, Maria Agudelo, John J. Cardeño, Carlos A. Rodriguez, Omar Vesga

**Affiliations:** 1 Department of Pharmacology and Toxicology, University of Antioquia Medical School, Medellín, Colombia; 2 Grupo Investigador de Problemas en Enfermedades Infecciosas, University of Antioquia Medical School, Medellín, Colombia; 3 Section of Infectious Diseases, Department of Medicine, Hospital Universitario San Vicente de Paul and University of Antioquia Medical School, Medellín, Colombia; Columbia University, United States of America

## Abstract

**Background:**

Drug regulatory agencies (DRA) support prescription of generic products of intravenous antibiotics assuming therapeutic equivalence from pharmaceutical equivalence. Recent reports of deaths associated with generic heparin and metoprolol have raised concerns about the efficacy and safety of DRA-approved drugs.

**Methodology/Principal Findings:**

To challenge the assumption that pharmaceutical equivalence predicts therapeutic equivalence, we determined in vitro and in vivo the efficacy of the innovator product and 20 pharmaceutically equivalent generics of gentamicin. The data showed that, while only 1 generic product failed in vitro (MIC = 45.3 vs. 0.7 mg/L, P<0.05), 10 products (including gentamicin reference powder) failed in vivo against *E. coli* due to significantly inferior efficacy (E_max_ = 4.81 to 5.32 vs. 5.99 log_10_ CFU/g, P≤0.043). Although the design lacked power to detect differences in survival after thigh infection with *P. aeruginosa*, dissemination to vital organs was significantly higher in animals treated with generic gentamicin despite 4 days of maximally effective treatment.

**Conclusion:**

Pharmaceutical equivalence does not predict therapeutic equivalence of generic gentamicin. Stricter criteria based on solid experimental evidence should be required before approval for human use.

## Introduction

There has been a dramatic increase in clinical use of generic medicines since 1980, but there are not systematic evaluations of their therapeutic efficacy compared with innovator products [1], [2]. Although essential to reduce health budget costs and to promote pharmaceutical competition and employment, generic drugs remain a topic of intense controversy as a result of the accelerated approval process for human use (which some disagree with), and sporadic reports of failures and deaths associated with their use [3]–[5]. There is an unsurpassable point in this controversy: forcing the manufacturers of generic drugs to go through the same process required to bring innovator drugs to market implies a cost overrun calculated in 150 to 800 million dollars that would hinder their mainstay objective, i.e., “to regulate and reduce the medicine's price” [6]. The solution has been a very short, straightforward and inexpensive process created to approve generic versions of innovator products, which requires no comparative preclinical or clinical safety and/or efficacy studies, hoping that generics would generate results similar to those obtained with the innovator drug [7]. In consequence, therapeutic equivalence is assumed after demonstration of pharmaceutical equivalence and bioequivalence with respect to a gold standard, usually the innovator [8]–[10]. Furthermore, only the active pharmaceutical ingredient (API) is considered responsible for pharmaceutical equivalence, without special attention to binders, diluents, excipients (fillers), impurities, and contaminants present in all formulations that may vary widely between generics and innovators affecting safety and efficacy [3], [11]–[13]. Of note, drug regulatory agencies (DRA) waive the requirement of bioequivalence for pharmaceutically equivalent intravenous solutions because their bioavailability is considered “self-evident” [14].

Aminoglycosides are concentration-dependent, highly bactericidal antibiotics that act mainly by inhibition of protein synthesis [15]. In many countries, gentamicin is the compound most frequently prescribed from this group to treat infections as serious as peritonitis, pyelonephritis, bacteremia, and endocarditis. Thus, a high number of generic versions are approved for clinical use, invariably without demonstration of in vivo efficacy or safety.

To find out if pharmaceutical equivalence is an adequate surrogate of therapeutic equivalence, we determined with the neutropenic mouse thigh infection model three pharmacodynamic outcomes (bactericidal efficacy, prevention of bacterial dissemination to distant vital organs, and toxicity), comparing 19 pharmaceutically equivalent generic products and the reference powder against the innovator of gentamicin. Significant inferiority in any of these endpoints was considered a therapeutic failure, i.e., lack of therapeutic equivalence [16].

## Materials and Methods

### Ethics Statement

The Animal Experimentation Ethics Committee of University of Antioquia approved and verified the fulfillment of the institutional policies.

### Bacteria and media

*Escherichia coli* SIG-1, a clinical strain, was the microorganism inoculated in the neutropenic mouse thigh infection model for all experiments with a microbiological endpoint (24 hours duration), and *Pseudomonas aeruginosa* GRP-0019, a fully-susceptible catheter isolate, for dissemination studies in the survival model (4 days long). The quality control organism for all susceptibility tests was *P. aeruginosa* ATCC 27853, as recommended by Clinical and Laboratory Standards Institute (CLSI) [17]. All bacteria were harvested in cation-adjusted Mueller-Hinton broth and agar for susceptibility tests and in trypticase soy broth and agar (Becton Dickinson, Sparks, MD) for in vivo studies. Trypticase soy agar supplemented with 5% sheep blood was used to recover the strains from ultra-freezing.

### Antimicrobial agents

All intravenous antibiotics were bought from reputable local drugstores and handled as instructed by the manufacturer. The innovator's brand-name was Garamicina® (GNT-S Plough, Schering-Plough SA, Bogota, Colombia) [18], and it was included in every experiment (in vitro and in vivo) to compare simultaneously with one or more generic products. The reference powder (not designed for human use) was imported directly from Sigma Aldrich (St Louis, MO, USA), and made part of the design as if it were a generic product. The study was designed to discriminate if generic failure in vivo was an isolated quality problem (one batch) or a constant phenomenon (any batch) independent of “quality” as it is understood by DRA worldwide. We divided generic products at random in two groups: Batch Group 1 had 10 products that were allocated to use the same batch for in vitro susceptibility testing and in vivo determination of efficacy, and Batch Group 2 had the remaining 10 products allocated to use different batches in vitro than in vivo. If generic failure was an incidental phenomenon, we should find a statistically significant difference (two-tailed Fisher's exact test) between Batch Groups 1 and 2 in terms of the number of products failing in vivo within each group. Table S1 details all products included in the study, their presentation, license number, maker, distributor, and the distribution of their batches for in vivo, in vitro, or both kinds of experiments.

### Susceptibility testing

Duplicates of minimal inhibitory (MIC) and bactericidal (MBC) concentrations of all products were determined twice (generics) or thrice (innovator product) by broth microdilution following CLSI methods [19]. GNT-Recipe was not commercially available when susceptibility testing was done, but one of GNT-Recipe batches (009040) from the same maker (Vitrofarma S.A.) and with the same license number (M-006660) was being distributed at the time as GNT-AZ pharma and was used for MIC and MBC determinations.

### Induction of neutropenia

Six-week-old, 23–27 g, female mice of the strain Udea:ICR[CD-1] were bred and housed in a murine pathogen-free barrier facility (Micro-Isolator System, Lab Products Inc., Seaford, DE, USA) with free access to sterile water plus vitamin K_3_ (Sigma-Aldrich Corp., St. Louis, MO, USA) and autoclaved mouse feed (Zeigler Bros. Inc., Gardners, PA, USA). Profound neutropenia (<10 neutrophils/mm^3^) was induced by injecting two doses of cyclophosphamide (Cytoxan®, Bristol-Myers Squibb, Mayaguez, Puerto Rico, USA) intraperitoneally 4 days (150 mg/kg) and 1 day (100 mg/kg) before infection [20].

### Induction of infection

Bacteria were resuscitated from ultra-freezing and grown in two steps to reach log-phase until OD_580_  =  0.3 (Spectro 22®, Labomed Inc., Culver City, CA, USA). Depending on the infecting microorganism, 10^6^ (*E. coli*) or 10^4^ (*P. aeruginosa*) CFU contained in 0.1 mL were injected into each thigh of anesthetized mice. The lower inoculum with *P. aeruginosa* was necessary to prevent premature death (before 12 hours) in the survival experiments, designed for a longer follow-up (4 days).

### *In vivo* pharmacodynamics (microbiological endpoint)

Neutropenic mice infected with *E. coli* SIG-1 were treated during 24 hours (h) using at least 5 total doses (TD) per product ranging from no effect (0.75) to maximum effect (768 mg/kg per day). Each TD was given to subgroups of 2 mice starting 2 h after inoculation (hour 0 for the model) and divided every 6 h (q6h) in 0.2-mL subcutaneous injections to optimize C_max_/MIC and AUC/MIC, the pharmacodynamic (PD) indices associated with efficacy in mice and humans with normal renal function [21], [22]. Untreated control mice were sacrificed for bacterial count at 3 time-points: just after inoculation (−2h), at the onset (0h), and at the end of therapy (24h). Treated mice were sacrificed at 24 h and their thighs dissected under aseptic technique, homogenized independently to a final volume of 10 mL of saline, serially diluted, plated and incubated at 37°C for 18 h under air atmosphere. The limit of detection was 100 CFU/thigh. Bacterial counts were stored in an Excel database (Microsoft, Seattle, WA) for subsequent analysis.

### Systemic bacterial dissemination and survival experiments (clinical endpoint)

To test if therapeutic inequivalence affects generics' ability to stop bacterial dissemination and eventually impact survival, we designed an experiment using 35 neutropenic mice randomly distributed in 3 groups: (1) infected with *P. aeruginosa* GRP-0019 in both thighs and mock-treated with sterile saline injections (survival controls, n = 5); (2) infected and treated (experimental group, n = 10 per product); and (3) non-infected but treated (toxicity controls, n = 5 per product). Treatment started either 2 (early) or 6 h (delayed treatment) after infection and consisted of generic (GNT-Recipe, Vitrofarma SA, Bogota, Colombia) or innovator gentamicin (GNT-S Plough) during 4 days, 768 mg/kg daily divided q6h in 0.2-mL subcutaneous injections. At the end, thighs, lungs, spleen and kidneys were aseptically removed and independently homogenized, diluted, plated, and incubated at 37°C for 18 h. In case of acute neuromuscular blockade after gentamicin injection, affected mice were treated with calcium gluconate (130 mg/kg), 1 to 3 subcutaneous doses until obtaining clinical response [23], [24]. Although calcium concentrations affect MIC and MBC determinations of aminoglycosides and these induce renal excretion of calcium, there is no evidence that calcium affects aminoglycoside pharmacokinetics or pharmacodynamics in vivo [25].

### Statistical analysis

For in vitro efficacy, the significance of the difference between geometric means of MIC and MBC was calculated with Kruskal-Wallis followed by Dunn's test (StatXact-5®, Cytel Software Corp., Cambridge, MA) [26]. The validation of a microbiological method to demonstrate the pharmaceutical equivalence of generic products of gentamicin is described thoroughly elsewhere [25]. The slopes and intercepts of concentration-response curves from each generic were compared against the innovator with the overall test for coincidence of regression lines.

For in vivo data, we calculated net antibacterial effect (E, dependent variable) of each product's dose (D, independent variable) by subtracting the number of CFU/g of untreated controls (hour 24) from CFU/g remaining in mice treated during 24 h (for this model, 1 thigh weighs 1 g). As E implies bacterial killing, it is a negative number, except under ineffective doses that eventually allow growth beyond that of untreated controls.

Least-squares nonlinear regression (NLR) was used to generate the sigmoid dose-response relationship typical of bactericidal antibiotics and described by the Hill's Equation, by which 
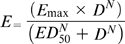
; *E* is the effect (dependent variable), *D* is the 24h total dose (independent variable), *E_max_* is the maximum effect and quantifies *efficacy* (the most important PD parameter), *ED_50_* is the dose yielding half the *E_max_* (a PD parameter measuring the drug's approximate *potency*), and *N* is the Hill's slope, the PD parameter describing the *affinity* between a drug and its target. In the system used here (the neutropenic mouse thigh infection model), *N* represents the steepness of the curve and reflects, although indirectly, the ability of the API to bind a specific molecular target [27]. These three are the primary PD parameters, all derived from the Hill's model (SigmaPlot 11.0®, SPSS Inc., Chicago, IL).

We also computed the doses (mg/kg per day) required in vivo to reach a net bacteriostatic effect (BD) and to kill the first, second and third logs of bacteria (1LKD, 2LKD and 3LKD respectively). These secondary PD parameters determine the *exact potency* of the antibiotic, and portray more clinical significance than ED_50_ because their magnitudes are corrected by the net bacterial growth (G) calculated from the number of microorganisms (CFU/g) growing in untreated controls (24h minus 0h) during the time of the experiment [22], [28]. Secondary PD parameters are expressed in the following equations:
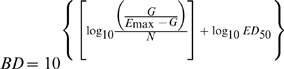
(1)

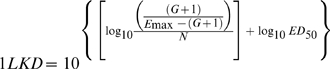
(2)

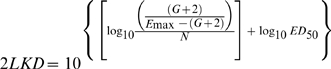
(3)

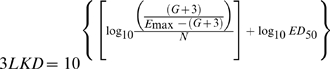
(4)The magnitude of significant primary and secondary PD parameters obtained for each generic product and the reference powder were compared with those obtained for the innovator by the overall test for coincidence of the NLR, a specialized statistical technique for Curve Fitting Analysis (CFA) (Prism 5.0, GraphPad Software, Inc., San Diego, CA) [29].

We used log-rank test for analysis of survival experiments and the Fisher's exact test followed by Bonferroni's inequality correction to determine differences between generic and innovator gentamicin to prevent bacterial dissemination to organs away from the infection site.

Although efficacy and bacterial dissemination were respectively the microbiological and clinical end-points of pharmacodynamic experiments, we also compared survival rate and frequency of adverse events despite the fact that the design had very low statistical power due to ethical considerations (i.e., to employ de minimum number of animals strictly necessary to demonstrate the principal endpoints). Thus, for the microbiological end-point (in vivo efficacy), accepting a 5% chance for a type I error (α-error) and expecting residuals' standard deviations <0.5 logs, the treatment of 10 animals per product to compare 20 generics (ANOVA) with the innovator confers 74% power to reject the null hypothesis (H*_0_*: generics  =  innovator product) if the magnitude of the difference in antibacterial efficacy is >1 log_10_ CFU/g. Likewise, for the clinical end-point (bacterial dissemination), accepting α = 0.05 and using a control to experimental subjects ratio of 0.5, a difference between generic and innovator of at least 66% (in protection from bacterial dissemination respect to untreated mice) would be necessary to assure 80% power [30]. For survival endpoints in experiments where treatment started 6 h post-infection and accepting α = 0.05, the use of 10 subjects per arm with an accrual interval of 3 h, an additional follow-up after the accrual interval of 96 h, a median survival time of 60 h for the arm treated with innovator, and a median survival time of 50 h for the arm treated with the generic, will confer only 6.2% power to detect differences in survival rate between both arms. For experiments where treatment started 2 h post-infection the median survival time of treated mice is not computable because mortality is far less than 50% (based on pilot experiments). Despite these obvious limitations in power, survival experiments with this design were approved and executed because pilot tests strongly suggested that using dissemination as their principal endpoint could generate enough power to demonstrate significant differences between both products.

## Results

### Antimicrobial agents

Seventeen of 21 products (81%) including the innovator were produced in Colombia (Table S1). One generic and the reference powder were made in USA; other 2 generic products were manufactured in Germany and Austria. It should be noted that Vitrofarma S.A. and Viteco S.A. (both from Bogota, Colombia) manufactured 7 (37%) and 5 (26%) of 19 generic products, respectively. We included all these products despite coming from the same maker because they were commercialized by different distributors and under different names. The results and conclusions of the study were not influenced at all by the preponderance of these two makers in the sample.

### Susceptibility testing

MIC and MBC (geometric mean and range) of each generic product, the reference powder and the innovator against *E. coli* SIG-1 or *P. aeruginosa* ATCC 27853 (quality control strain) are shown in Table 1. Statistically significant differences were observed only for generic GNT-AZ pharma (batch 009040), which displayed very high MIC and MBC (45.25 and 64 mg/L, respectively) compared with the innovator (0.71 and 0.79 mg/L, respectively, P<0.05 in both cases). The MBC to MIC ratio of this product remained undistinguishable from the innovator (P = 0.347). This batch of GNT-AZ pharma was studied in the animal model under the name GNT-Recipe.

**Table 1:** Minimal inhibitory (MIC) and bactericidal (MBC) concentrations (geometric mean and range) and MBC/MIC ratios of 19 generic products of gentamicin (GNT), the reference powder, and the innovator against *E. coli* SIG-1 (clinical strain) and *P. aeruginosa* ATCC 27853 (control strain) by standard broth microdilution techniques.

	*Escherichia coli* SIG-1						*Pseudomonas aeruginosa* ATCC 27853					
GNT Product	MIC (mg/L)			MBC (mg/L)			MIC (mg/L)			MBC (mg/L)		
	Mean	Min	Max	Mean	Min	Max	Mean	Min	Max	Mean	Min	Max
**S Plough**	0.71	0.50	1.00	0.79	0.50	2.00	1.26	0.50	4.00	2.52	1.00	8.00
**Abbott**	0.84	0.50	1.00	2.00	1.00	4.00	1.19	1.00	2.00	4.76	4.00	8.00
**Anglopharma**	0.84	0.50	2.00	1.41	0.50	4.00	1.41	1.00	2.00	3.36	2.00	8.00
**Az pharma** *	45.25	32.0	64.0	64.00	64.0	64.0	45.25	32.0	64.0	64.00	64.0	64.00
**Biochemie**	1.19	0.50	2.00	1.41	1.00	2.00	1.19	1.00	2.00	4.00	2.00	8.00
**Biogenta**	0.59	0.25	1.00	1.00	0.50	2.00	1.19	1.00	2.00	4.00	2.00	8.00
**Colmed**	0.59	0.50	1.00	1.19	0.50	4.00	0.84	0.50	1.00	2.83	2.00	8.00
**Gencol**	0.84	0.50	2.00	1.00	0.50	4.00	1.41	1.00	2.00	4.76	2.00	16.00
**Genfar**	1.00	0.50	2.00	1.68	0.50	4.00	2.00	2.00	2.00	4.00	4.00	4.00
**Lab America**	1.19	1.00	2.00	2.00	1.00	4.00	1.68	1.00	2.00	4.76	2.00	8.00
**Labinco**	1.00	0.50	2.00	1.41	1.00	2.00	1.41	1.00	2.00	4.76	2.00	8.00
**La Sante**	0.71	0.50	1.00	1.00	0.50	2.00	1.00	1.00	1.00	4.76	2.00	8.00
**Memphis**	1.19	0.50	2.00	1.68	0.50	4.00	1.19	0.50	2.00	3.36	2.00	4.00
**Merck**	0.84	0.50	2.00	0.84	0.50	2.00	1.41	1.00	2.00	2.83	2.00	4.00
**MK**	0.59	0.50	1.00	0.71	0.50	2.00	1.19	1.00	2.00	2.00	2.00	2.00
**Ophalac**	1.00	0.50	2.00	2.83	2.00	4.00	1.68	1.00	2.00	4.76	4.00	8.00
**Pentacoop**	1.19	0.50	2.00	2.00	1.00	4.00	2.00	2.00	2.00	4.76	4.00	8.00
**Rande**	1.19	0.50	2.00	1.68	1.00	2.00	1.41	1.00	2.00	4.00	2.00	8.00
**Servipharm**	1.41	1.00	2.00	4.00	2.00	8.00	1.41	1.00	2.00	4.00	2.00	8.00
**Sigma**	0.56	0.25	1.00	0.79	0.25	2.00	1.00	1.00	1.00	2.25	1.00	4.00

* P-value <0.05 by Dunn's multiple comparison test. This product (GNT-AZ pharma batch 009040) was commercialized later as GNT-Recipe (batch 009040) and therefore tested in the animal model, failing that test too.

MICs, MBCs and MBC/MIC ratios of all other gentamicin generic products against *E. coli* were not different to those of the innovator (Table 1). Quality control results with *P. aeruginosa* and innovator gentamicin were always located within the range accepted by CLSI (0.25–4 mg/L).

### Pharmaceutical equivalence

Generic products of gentamicin had in vitro the same potency and concentration of the innovator, i.e., they were all pharmaceutical equivalents. Details of these data can be seen in a previous publication [25]. GNT-Anglopharma, GNT-Merck, GNT-Rande, and GNT-Servipharma were not available at the time of these tests. Batch 009040 (GNT-AZ pharma and GNT-Recipe) was not available either, but other batches from both distributors were included and found pharmaceutically equivalent to GNT-S Plough [25].

### *In vivo* pharmacodynamics

**Figure 1:**
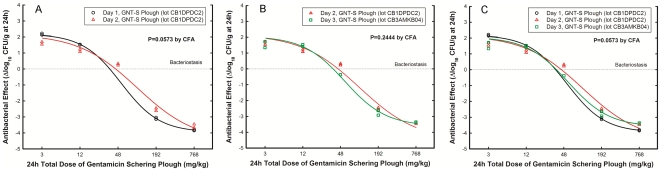
Repeatability of the animal model to determine therapeutic equivalence of generic gentamicin products. The graphs illustrate the non-significant differences on the innovator's pharmacodynamic profiles when a single (within-batch variation, Panel A), or a second (between-batch variation, Panel B), or multiple lots (total variation, Panel C) are used to treat *E. coli* SIG-1 in vivo. In all graphs showing data obtained with the animal model every data-point represents one mouse (mean of two thighs; if standard errors of the mean were to be included, the bars would not be longer than 0.5 logs).

Mice had 10^7.24–7.57^ and 10^9.44–9.68^ CFU per thigh before starting and after ending therapy, respectively (net growth range of *E. coli* SIG-1  =  2.00–2.26 log_10_ CFU/g in 24 h). No significant variation in net growth was observed between untreated controls along all the experiments performed (P = 0.415). Figure 1 shows the reproducibility of the animal model; for this, we compared the pharmacodynamic profiles derived from three non-linear regressions computed for the innovator product when we used the same (within-batch variation, CB1DPDC2 in day 1 vs. CB1DEDC2 in day 2, P = 0.0573; panel A), or different batches (between-batch variation, CB1DPDC2 in day 2 versus CB3AMKB04 in day 3, P = 0.2444; panel B), or all batches from GNT-S Plough studied in vivo (P = 0.0573; panel C).

Reproducibility of the animal model was also evident in its capacity to consistently detect generic failure independently of the product or batch involved. Table 2 shows that there was no difference (P = 0.6563 by Fisher's exact test) between Batch Groups 1 and 2, demonstrating that in vivo failure of generic products of gentamicin was detected the same if one batch was tested in vivo and vitro (60% failed) or if different batches were used in each test (40% failed). Table 2 also shows that in vitro testing is not sensible to detect absence of therapeutic equivalence (despite duplicate broth microdilution assays repeated two of three times), while the animal model was able to detect the only product that failed in vitro (batch 009040 from GNT-AZ pharma and GNT-Recipe) plus all others that were indistinguishable by conventional MIC and MBC testing.

**Table 2:** Randomization of generic products in two groups to establish if in vivo failure was incidental (one batch) or constant (any batch): identification of non-therapeutically equivalent generics is independent of the batches of each product studied in vitro and/or in vivo.

		Generic Product Failure	
Batch Group	Gentamicin Generic Product	In vitro	In vivo
1	Abbott	No	Yes
(same batch tested in vitro and in vivo)	Biochemie	No	Yes
	Colmed	No	Yes
	Gencol	No	Yes
	Lab America	No	No
	Merck	No	Yes
	Ophalac	No	No
	Rande	No	No
	Servipharm	No	No
	Sigma	No	Yes
**2 **	**Anglopharma**	**No**	**Yes**
**(different batches tested in vitro and in vivo)**	**Az pharma** *	**Yes** *	**No**
	**Biogenta**	**No**	**No**
	**Genfar**	**No**	**No**
	**Labinco**	**No**	**Yes**
	**La Sante**	**No**	**No**
	**Memphis**	**No**	**No**
	**MK**	**No**	**Yes**
	**Pentacoop**	**No**	**No**
	**Recipe** *	**Yes**	**Yes†**

*This product (AZ pharma batch 009040) was commercialized later as Recipe (batch 009040) and therefore tested in the animal model, failing that test too (†). However, AZ pharma batches 303030 (in vitro) and 0110059 (in vivo) did not fail; (please see Discussion).

Table 3 shows primary (E_max_, ED_50_, N) and secondary (BD, 1LKD, 2LKD, 3LKD) pharmacodynamic parameters of all generics compared with the innovator. All gentamicin products fitted Hill's model, which described their dose-effect relationships with an excellent fit, generating multicollinearity-free, highly significant PD parameters, and a nonlinear regression fulfilling the assumptions of normality, constant variance and independence (Table 3).

**Table 3:** In vivo efficacy of 19 generic products, the reference powder and the innovator of gentamicin (simple nonlinear regression analysis under Hill's model; all products passed normality and constant variance tests).

Gentamicin	Adj. R^2^	E_max_	SE	ED_50_	SE	N	SE	BD	SE	1LKD	SE	2LKD	SE	3LKD	SE	P value (CFA)
S Plough*	0.97	5.99	0.23	55.75	6.44	1.20	0.12	33.77	2.67	59.71	4.45	107.43	9.52	243.05	29.41	NA
Abbott	0.98	5.21	0.11	27.26	2.47	1.99	0.25	22.65	2.02	33.51	2.87	53.62	5.80	NS	-	0.001
Anglopharma	0.90	4.81	0.30	27.82	6.92	1.67	0.54	24.03	5.50	40.11	9.80	80.43	28.97	NS	-	0.022
AZ pharma	0.98	5.45	0.26	58.04	7.43	1.59	0.29	44.80	4.27	71.43	7.58	122.00	18.18	365.41	141.17	0.187
Biochemie	0.98	4.86	0.15	38.14	4.25	1.42	0.18	31.45	2.99	56.79	5.71	124.69	17.37	NS	-	0.001
Biogenta	0.98	6.44	0.42	74.87	13.56	1.06	0.15	40.09	3.44	73.52	6.42	134.31	15.31	285.50	42.63	0.577
Colmed	0.99	5.21	0.13	27.73	2.24	1.82	0.20	21.56	1.63	33.04	2.36	54.00	4.90	165.81	50.94	0.004
Gencol	0.96	5.26	0.37	59.55	11.72	1.43	0.33	45.49	6.29	77.91	12.14	147.38	31.56	NS	-	0.043
Genfar	0.99	5.40	0.16	37.49	3.03	1.86	0.26	28.80	2.38	43.17	3.05	67.76	6.40	157.75	33.38	0.144
Lab America	0.98	5.91	0.29	47.33	7.12	1.21	0.17	29.91	3.18	53.00	5.53	96.67	12.68	232.64	44.94	0.814
Labinco	0.99	4.84	0.09	36.07	1.89	2.27	0.26	31.41	1.76	45.48	2.07	73.73	5.42	NS	-	0.004
La Sante	0.99	5.71	0.23	43.21	5.08	1.43	0.21	28.82	2.84	47.59	4.35	80.77	9.51	179.79	33.20	0.639
Memphis	0.99	5.55	0.21	52.94	5.47	1.55	0.22	36.76	3.07	59.06	4.80	98.16	10.87	222.54	40.48	0.479
Merck	0.94	5.07	0.23	27.42	5.14	1.61	0.38	21.24	3.75	34.98	6.09	63.30	14.62	NS	-	0.025
MK	0.99	5.10	0.14	26.18	2.44	1.66	0.19	20.20	1.67	32.64	2.70	57.52	6.34	NS	-	0.004
Ophalac	0.98	6.46	0.63	97.56	29.2	0.93	0.16	45.59	5.69	91.36	11.33	180.73	25.78	416.96	71.50	0.067
Pentacoop	0.98	5.68	0.27	38.50	5.82	1.32	0.21	27.13	3.21	46.59	5.24	84.56	11.25	231.68	50.87	0.258
Rande	0.99	6.02	0.16	46.35	4.09	1.09	0.08	28.32	1.78	52.82	3.08	101.76	6.78	261.75	23.48	0.392
Recipe	0.99	5.32	0.13	48.53	4.28	1.22	0.11	34.62	2.45	64.45	4.59	132.45	12.23	672.49	220.61	0.022
Servipharm	0.97	5.56	0.32	41.25	7.56	1.24	0.22	29.37	3.88	52.94	7.33	103.05	18.76	NS	-	0.519
Sigma	0.97	5.07	0.29	72.71	10.94	2.19	0.54	59.49	6.38	85.77	11.95	132.01	25.78	NS	-	0.000

*Innovator product; Adj R^2^: adjusted coefficient of determination; E_max_: maximum effect; SE: standard error; ED_50_: effective dose to kill 50% of E_max_; N: slope; BD: bacteriostatic dose; 1LKD, 2LKD, 3LKD: 1, 2, or 3 log kill dose, respectively; CFA: curve fitting analysis; NS: parameter value not significantly different from zero.

**Figure 2:**
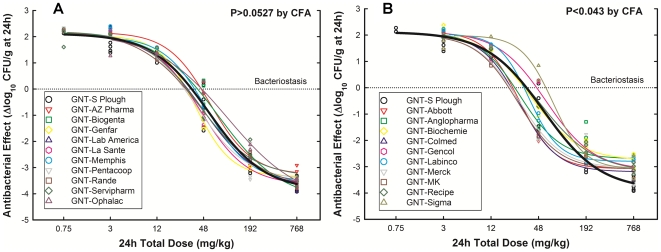
In vivo pharmacodynamics. Panels illustrate the dose-response curves derived from the neutropenic mouse thigh infection model of 19 generic products and the reference powder with (A) or without (B) therapeutic equivalence respect to the innovator.

**Figure 3:**
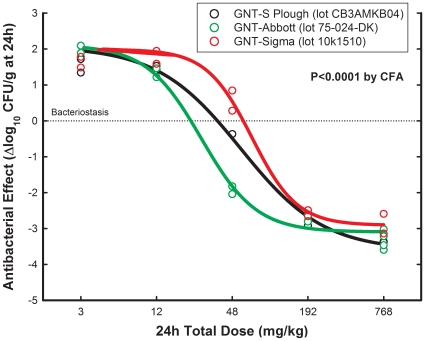
Unpredictability of therapeutic equivalence from pharmaceutical equivalence. The graph illustrates the dose-response curves of gentamicin made by three well-reputed makers: Abbott, Sigma and S. Plough. Abbott and Sigma were indistinguishable from S Plough in terms of concentration and potency of the active pharmaceutical ingredient, MIC, MBC, MBC/MIC ratios but significantly different in terms of therapeutic efficacy, although the same batch of each product was tested in vitro and in vivo.

From 20 generic products, 10 had PD profiles not significantly different from the innovator (Fig. 2, panel A) and 10 (including the reference powder) displayed significantly lower efficacy (Fig. 2, panel B), killing approximately 1 million less bacteria per gram of tissue than the innovator. In vivo failure happened independently of the maker (or its prestige), the vendor, the country of manufacture, pharmaceutical equivalence, or MBC and MIC identity with GNT-S Plough. Figure 3 illustrates the highly significant difference (P<0.0001) in PD profiles of two generics from very well reputed makers, one destined to clinical use (GNT-Abbott) and the other to experimental use (GNT-Sigma), with GNT-S Plough; all three products had the same batch studied in vitro and in vivo, illustrating how pharmaceutical equivalence cannot predict therapeutic equivalence.

In vivo potency was computed in the form of primary (ED_50_) and secondary PD parameters because it is a reliable estimate of the dose required in vivo to reach specific endpoints, such as bacteriostasis (BD) or bactericidal effect of one (1LKD) or more (2LKD, 3LKD) logs of microorganisms per gram of tissue. GNT-S Plough, for example, demonstrated no difference in potency along all experiments: as expected when there are no differences in the comparison of the complete nonlinear regression curves (Figure 1, panel C), ED_50_ was indistinguishable within or between lots of GNT-S Plough (ED_50_±SEM: batch CB1DPDC2 in day 1  =  53.7±2.9, batch CB1DPDC2 in day 2  =  96.5±38.1, batch CB3AMKB04 in day 3  =  48.0±7.5 mg/kg per day; P = 0.0640 by CFA). Although 6 of 10 failing products appear more potent than the innovator under simple NLR (Table 3), in all cases it is a false impression given by their lack of bactericidal efficacy. Multiple NLR (Table 4) allows computation of ED_50_ and N for each product assuming that the null hypothesis is correct, i.e., that all generics are as effective as the innovator. For example GNT-Abbott (Table 3), a less effective but apparently more potent generic, was in fact not more potent when required to reach the innovator's efficacy under multiple NLR (3LKD  =  241±28.3 vs. 203±45.9 mg/kg per day, respectively; P  = 0.511 by CFA). The same conclusion was reached when identical analysis was applied to any other inequivalent product apparently more potent than GNT-S Plough: GNT-Abbott, GNT-Colmed, GNT-Merck and GNT-MK, P = 0.067 by CFA of ED_50_.

**Table 4:** Pharmacodynamic parameters obtained by multiple NLR of 10 generic products of gentamicin with significantly lower efficacy than the innovator.

GENTAMICIN	NORM.	CV	ED_50_	SE	N	SE
**S Plough**	OK	OK	55.75	7.56	1.20	0.15
**Abbott**	Failed	OK	39.90	6.27	1.08	0.15
**Anglopharma**	OK	OK	60.91	13.86	0.80	0.12
**Biochemie**	OK	OK	74.35	12.59	0.88	0.10
**Colmed**	OK	OK	38.36	5.69	1.20	0.18
**Gencol**	OK	OK	79.07	13.24	1.05	0.15
**Labinco**	OK	OK	56.37	9.73	1.14	0.19
**Merck**	OK	OK	47.34	9.01	0.91	0.12
**MK**	OK	OK	40.28	6.72	1.03	0.15
**Recipe**	OK	OK	69.51	10.95	0.91	0.10
**Sigma**	OK	OK	99.65	14.70	1.25	0.17

Under this analysis, maximum effect (E_max_) for all products is fixed to the innovator's (E_max_ = 5.99 log_10_ CFU/g).

Norm: normality test; CV: Constant variance test; ED_50_: effective dose to kill 50% of E_max_; N: Hill's slope; SE: standard error.

Hill's slope (N) is the primary PD parameter that measures the sensitivity with which a particular system translates the concentration of a drug into an effect; therefore, the highly specific mechanism of action of gentamicin usually gives N near to 1, just as we observed with the innovator (N = 1.20±0.2) or the therapeutically equivalent generics (N = 1.30±0.19; P = 0.1846 by CFA). In contrast, the 10 products without therapeutic equivalence exhibited slopes significantly higher than the innovator (1.73±0.3; P = 0.0261).

### Systemic bacterial dissemination and survival experiments

**Figure 4:**
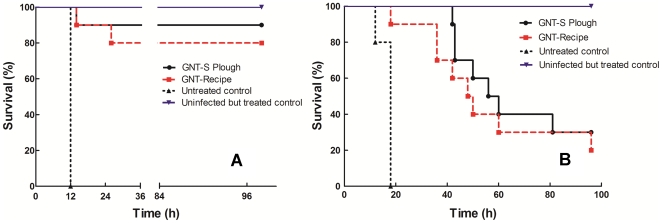
Results from survival experiments. Log-rank test curves obtained from neutropenic mice infected in the thighs with *P. aeruginosa* GRP-0019 and treated during 4 days with placebo (n = 5), GNT-Recipe (n = 10), or the innovator of gentamicin (n = 10) at the dose required for maximal effect (768 mg/kg per day divided q6h), starting 2 h (panel A) or 6 h (panel B) post-infection. Uninfected neutropenic mice serving as toxicity controls received the same treatment and were identical to the other animals but, instead of *P. aeruginosa*, were mock-inoculated in the thighs with sterile saline (n = 5 mice per gentamicin product). No significant impact on survival was detected between both gentamicin products.

Figure 4 shows survival rates for infected but untreated mice (survival controls), infected and treated mice (experimental group), and non-infected but treated mice (toxicity controls). All untreated mice died of sepsis between 12 and 18 h after infection, while most of those receiving early treatment (2 h after infection) survived the experiment in good health without difference between generic (80%) and innovator gentamicin (90% survival, P = 0.557).

**Figure 5:**
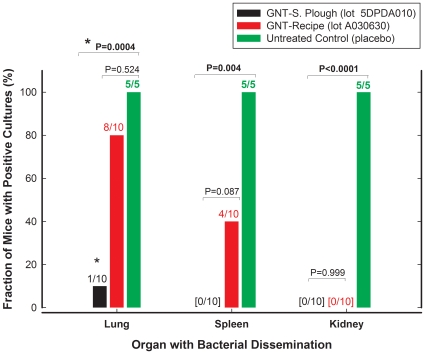
Bacterial dissemination to distant vital organs during survival experiments. After the fourth day of treatment (same experimental design described in Figure 4) the animals that survived were euthanized and their thighs, lungs, spleens and kidneys processed for bacterial counting (mice found dead were processed immediately). Although thigh counts are not illustrated in the graph, GNT-Recipe left >100,000 CFU/thigh in 80% of the animals, not different from placebo treatment (100%), but significantly different from GNT-S Plough, the innovator product (P<0.0001).

Late treatment (6 h after infection) was associated with lower survival than early treatment but, again, no differences in survival were detected between generic (20%) and innovator gentamicin (30%, P = 0.448). Despite this apparent similarity in survival, a great difference was evident in the capacity of both products to sterilize the thighs of survivors after 4 days of treatment: 100% mice in the innovator group had sterile thighs, while 0% achieved this goal after generic treatment, leaving approximately 4 log_10_ CFU of *P. aeruginosa* per thigh. Additional differences were observed between products in the rate and extent of bacterial dissemination to different organs (lungs, spleen and kidneys); treatment with generic gentamicin could not prevent lung dissemination of *P. aeruginosa* (80%), a result that was undistinguishable from placebo (100% of mice had positive lung cultures, P = 0.5238). The innovator, on the other hand, did protect mice from lung dissemination (10% positive lung cultures, P = 0.0004 compared with generic or placebo treatment by Bonferroni inequality test). Spleen dissemination was 0%, 40%, and 100% for innovator, generic, and the untreated control group, respectively, but the difference between innovator and generic did not reach statistical significance (P = 0.0867). Kidneys dissemination, as expected from an antibiotic with renal clearance, was completely prevented by both generic and innovator gentamicin (Figure 5). Even though acute neuromuscular blockade was twice as frequent after injection of generic (13 events) than innovator gentamicin (6 events), it did not reach statistical significance (P = 0.158).

## Discussion

Generic substitution for brand-name antibiotics has become an accepted and widespread practice, and bioequivalence remains the mainstay criterion to gain the public trust in such strategy. The methodology for testing generics and the parameters accepted as proof of bioequivalence have been established by each national DRA [25], [31]–[33], but all exempt parenteral solutions from demonstrating this equivalence because it is considered “self-evident” [25]. Some authors have expressed concerns about this approach, especially for drugs with narrow therapeutic index, but no attempt has been done to challenge this dogma [34]–[36]. Here, we demonstrated that even pharmaceutically equivalent products of gentamicin may fail therapeutic equivalence compared with the innovator in the same animal model used in preclinical studies [21].

In open contradiction with pharmaceutical equivalence, 53% of 19 generic products and the reference powder (GNT-Sigma) were devoid of therapeutic equivalence. The magnitude of this inequivalence (and its real-life relevance and significance) can be estimated from the lack of efficacy (all 10) or potency (6 products): ineffective products killed per gram of tissue 0.77–0.91 million less bacterial cells, and required 2.77× to >3.16× greater dose to kill the third log of bacteria compared with the innovator. This lack of potency is critical for an aminoglycoside, because we found during standardization of the survival model that the dose required for maximal efficacy (768 mg/kg per day) was neurotoxic for the mice if given for more than 24 h, and a dose twice that (1536 mg/kg) was lethal. Bacterial dissemination from the thighs to distant organs like the lungs also supports the argument in favor of the relevance of the differences demonstrated by the animal model. Inequivalent generics were not only incapable of sterilizing (detection limit, 100 CFU/thigh) the site of infection after 4 days of treatment at the dose required for maximal efficacy, but left 100,000 bacterial cells per thigh that disseminated effectively to the lungs (8-fold compared with the innovator). In burnt patients, for example, 1000 cells of *P. aeruginosa* are enough to invade the skin [37].

Although we observed inferior efficacy (E_max_), less potency (3LKD), altered target-affinity (N), higher frequency of bacterial dissemination to different organs, and twice more adverse events (neuromuscular blockade), our experimental design did not have enough power to detect significant impact on survival: 770 mice would be necessary per arm to obtain a power of 80% to demonstrate a difference of 10 h in the median time-to-death. Translating this situation to clinical practice would imply the exposure of thousands of patients to demonstrate any difference in survival after exposure to generic gentamicin, and explains the absence of therapeutic failures associated with use of “bioequivalent” generics [38]. It does not imply that generic use is devoid of consequences, it just confirms that mortality is an exceedingly gross outcome to compare efficacy in vivo. In a recent cohort study, Ammerlaan et al. found that there are no differences in efficacy and 30-day mortality rates between inadequate or adequate therapy for patients with *S. aureus* bacteremia [39]. We also believe that these findings reflect that mortality is a very crude measure to show differences in effectiveness of antibiotic therapy [40].

The absence of in vitro differences between generics and innovator confirms that these products were quality-drugs if judged by current regulations and requirements. Batch 009040 was unique in that it (as GNT-AZ pharma) failed in vitro (MIC 36–64× and MBC 25–36× higher than GNT-S Plough against *P. aeruginosa* and *E. coli*) and in vivo (as GNT-Recipe), leaving alive 768,000 *E. coli* cells per gram of tissue in comparison with the innovator. The other 9 inequivalent generics failed in vivo despite being equivalent by MIC, MBC, MBC/MIC ratios, concentration and potency of API, and independently of their origin (generics imported from Austria, Germany and USA invariable failed therapeutic equivalence). It is then clear that the majority (90%) of nonequivalent generics did not have quality problems based on current regulations, the real problem originates in a systematic failure to establish true bioequivalence: current tests do not predict in vivo efficacy, and the term bioequivalence undoubtedly implies much more than similar pharmacokinetics.

Panels A and C of Figure 1 show that two of three PD profiles of the innovator almost reach a statistically significant difference (P = 0.0573). The high variance depends on batch CB1DPDC2 compared against itself in two different experiments (called day 1 and day 2), and is explained by a very high efficacy (E_max_ = 6.737±0.9257 log_10_ CFU/g) with a large standard error for that parameter. Since ED_50_ computation depends on the E_max_, it is also high and with a large error (96.47±38.1 mg/kg per day), and such influence is confirmed by two relatively large variance inflation factors (9.9941, 7.9508 and 3.2973 for E_max_, ED_50_ and N), indicating the presence of a mild multicollinearity between the first two PD parameters. If gentamicin products are compared with the innovator using only the dose-response curves obtained during the experiment in which generic was studied, 3 more generics would appear failing in vivo, for a total of 13. Our design is to compare all generics against the NLR of all the data from the innovator, a less powerful but more reliable strategy in that all the variance of the data obtained with the innovator is taken into account. Given that the difference between the individual NLRs of GNT-S Plough does not reach significance, all the data belong to the same population and therefore are better described by a single dose-response curve, i.e., a significantly more robust and reliable NLR. It prevents the model from identifying as failures those generics with borderline differences respect to the comparator product.

Our results suggest that excipients and impurities play a key role in efficacy [3], [11], [13]. GNT-Sigma, the reference powder, is a pure, analytical-grade product, devoid of excipients (therefore not intended for clinical use), that displayed pharmaceutical equivalence and similar in vitro activity, but failed in vivo by a large difference in efficacy compared with the innovator (E_max_ = 5.07±0.29 vs. 5.99±0.23 log_10_ CFU/g, respectively). GNT-Sigma required greater doses for bacteriostasis and to kill the first and second logs of bacteria (BD, 1LKD, and 2LKD were 1.76×, 1.44×, and 1.23× greater, respectively), and could not kill 3 logs at maximal effect. Clinical-grade generic products that failed in vivo displayed the same PD pattern. Under current regulations, the innovator is required to publish the chemical structure of its molecule, but not the excipients of its pharmaceutical product [8], [9], [14], [33]. Only recently, the British, Chinese and United States pharmacopeias accepted that the presence of these unknown or unwanted chemicals even in small amounts may influence the efficacy and safety of the API [12], [31], [41], [42]. This change of policy might have been precipitated by the recent demonstration of numerous fatalities (thousands) associated with the use of a contaminated generic heparin in America, Australia and Europe [3]. The fact that GNT-Sigma had one of the worst efficacies despite its high-quality grade, points towards the absolute lack of excipients as the cause for its ineffectiveness. These results reflect the importance of excipients for efficacy, and suggest that failure of generic gentamicin might be related to the use of excipients or the presence of impurities not present in the innovator product. It is well-known that excipients are promoters of degradation more than stabilizers of drug substances, but their role depends on all factors that may cause the molecular transformation of the drug, including the interaction between molecules adjacent to the API, as is often the case with impurities and excipients [43], [44]. Then, suboptimal excipients or impurities may induce changes that compromise the clinical efficacy and safety (toxicity), especially if they have the ability to interact and modify the API promoting an unexpected instability of the generic antibiotic [45]–[47]. Evidence in favor of this hypothesis was recently published by Mastoraki et al, who were intrigued by an unexpectedly high incidence of post-operative infections following a change from innovator to generic cefuroxime for surgical prophylaxis of coronary artery bypass patients. They found that the generic formulation was hydrolyzed after reconstitution in a much shorter time compared with the innovator, breaking down into two ineffective parts [5], [48].

Aminoglycosides target a conserved region of rRNA sequence discriminating between prokaryotic and eukaryotic ribosomes. Their highly specific action is related to the unique presence in 16S rRNA of an A1408·A1493 base pair (*E. coli*) instead of the G1408:A1493 base pair on human's ribosome [49]. The geometry of the A·A pair is different to the G·A pair, creating a binding pocket with different affinity for the ring I of aminoglycosides, more favorable to the interaction of the antibiotic with the prokaryotic ribosome. According with this, antibiotic resistance could result from either decreased affinity of the antibiotic for the ribosome or non-productive binding of the drug to ribosome. Although we used a fully susceptible strain, some pharmaceutical equivalent generics failed efficacy, suggesting an alteration in the affinity or the binding. The greater Hill slopes of inequivalent generics could suggest the presence of other components like excipients or impurities interfering with API, reducing its affinity for or inducing non-productive binding to active ribosomes.

In conclusion, therapeutic equivalence cannot be reliably predicted from pharmaceutical equivalence of gentamicin generic products. A thorough revision of current regulations, including the development and application of appropriate preclinical tests to determine therapeutic equivalence, should be considered before approval of generic gentamicin for human or veterinary use.

## Supporting Information

Table S1 Detailed information of the products studied, their sources, and batches employed *in vitro* and *in vivo*.
(0.05 MB PDF)

## References

[1] Henry D, Lexchin J (2002). The pharmaceutical industry as a medicines provider.. Lancet.

[2] Kirking DM, Ascione FJ, Gaither CA, Welage LS (2001). Economics and structure of the generic pharmaceutical industry.. J Am Pharm Assoc (Wash ).

[3] Blossom DB, Kallen AJ, Patel PR, Elward A, Robinson L (2008). Outbreak of adverse reactions associated with contaminated heparin.. N Engl J Med.

[4] Feldschreiber P (2009). Public health issues with counterfeit medicines.. Clin Med.

[5] Mastoraki E, Michalopoulos A, Kriaras I, Mouchtouri E, Falagas M (2008). Incidence of postoperative infections in patients undergoing coronary artery bypass grafting surgery receiving antimicrobial prophylaxis with original and generic cefuroxime.. J Infect.

[6] Katzung BG (2007). Basic & clinical pharmacology.

[7] Henderson JD, Esham RH (2001). Generic substitution: issues for problematic drugs.. South Med J.

[8] Meredith P (2003). Bioequivalence and other unresolved issues in generic drug substitution.. Clin Ther.

[9] Nation RL, Sansom LN (1994). Bioequivalence requirements for generic products.. Pharmacol Ther.

[10] Strom BL (1987). Generic drug substitution revisited.. N Engl J Med.

[11] Apte SP, Ugwu SO (2003). A Review and Classification of Emerging Excipients in Parental Medications.. Pharmaceutical Technology Europe.

[12] Dodd S, Besag FM (2009). Lessons from contaminated heparin.. Curr Drug Saf.

[13] Roy J (2002). Pharmaceutical impurities–a mini-review.. AAPS PharmSciTech.

[14] Welage LS, Kirking DM, Ascione FJ, Gaither CA (2001). Understanding the scientific issues embedded in the generic drug approval process.. J Am Pharm Assoc (Wash ).

[15] Gonzalez LS, Spencer JP (1998). Aminoglycosides: a practical review.. Am Fam Physician.

[16] Rheinstein PH (1990). Therapeutic inequivalence.. Drug Saf.

[17] Clinical and Laboratory Standards Institute (2003). Methods for dilution antimicrobial susceptibility test for bacteria that grow, approved standard M7-A6.

[18] Weinstein MJ, Luedemann GM, Oden EM, Wagman GH, Rosselet JP (1963). Gentamicin, a new antibiotic complex from Micromonospora.. J Med Chem.

[19] Clinical and Laboratory Standards Institute (2006). Performance standards for antimicrobial susceptibility testing, approved standard M100-S16.

[20] Zuluaga AF, Salazar BE, Rodriguez CA, Zapata AX, Agudelo M, Vesga O (2006). Neutropenia induced in outbred mice by a simplified low-dose cyclophosphamide regimen: characterization and applicability to diverse experimental models of infectious diseases.. BMC Infect Dis.

[21] Craig WA (1998). Pharmacokinetic/pharmacodynamic parameters: rationale for antibacterial dosing of mice and men.. Clin Infect Dis.

[22] Gerber AU, Craig WA, Brugger HP, Feller C, Vastola AP (1983). Impact of dosing intervals on activity of gentamicin and ticarcillin against Pseudomonas aeruginosa in granulocytopenic mice.. J Infect Dis.

[23] Brashier MK, Geor RJ, Ames TR, O'Leary TP (1998). Effect of intravenous calcium administration on gentamicin-induced nephrotoxicosis in ponies.. Am J Vet Res.

[24] Singh YN, Harvey AL, Marshall IG (1978). Antibiotic-induced paralysis of the mouse phrenic nerve-hemidiaphragm preparation, and reversibility by calcium and by neostigmine.. Anesthesiology.

[25] Zuluaga AF, Agudelo M, Rodriguez CA, Vesga O (2009). Application of microbiological assay to determine pharmaceutical equivalence of generic intravenous antibiotics.. BMC Clin Pharmacol.

[26] Tracy M, Wanahita A, Shuhatovich Y, Goldsmith EA, Clarridge JE (2001). Antibiotic susceptibilities of genetically characterized Streptococcus milleri group strains.. Antimicrob Agents Chemother.

[27] Toutain PL, Lees P (2004). Integration and modelling of pharmacokinetic and pharmacodynamic data to optimize dosage regimens in veterinary medicine.. J Vet Pharmacol Ther.

[28] Lees P, Cunningham FM, Elliott J (2004). Principles of pharmacodynamics and their applications in veterinary pharmacology.. J Vet Pharmacol Ther.

[29] Glantz SA (2006). Primer of Biostatistics.

[30] Dupont WD, Plummer WD (1997). PS power and sample size program available for free on the internet.. Controlled Cllin Trials.

[31] British Pharmaceutical Society (2007). British Pharmacopoeia.

[32] Food and Drug Administration (2001). Guidance for Industry: Bioanalytical Method Validation.

[33] U.S.Department of Health and Human Services, Center for Drug Evaluation and Research, Food and Drug Administration (FDA) (2001). Guidance for industry. Statistical Approach to Establishing Bioequivalence.

[34] Generic Drugs (2002). Med Lett Drugs Ther.

[35] Dettelbach HR (1986). A time to speak out on bioequivalence and therapeutic equivalence.. J Clin Pharmacol.

[36] Jones RN, Fritsche TR, Moet GJ (2008). In vitro potency evaluations of various piperacillin/tazobactam generic products compared with the contemporary branded (Zosyn, Wyeth) formulation.. Diagn Microbiol Infect Dis.

[37] Pollack M (1984). The virulence of Pseudomonas aeruginosa.. Rev Infect Dis.

[38] Rodriguez CA, Agudelo M, Cataño JC, Zuluaga AF, Vesga O (2009). Potential Therapeutic Failure of Generic Vancomycin in a Liver Transplant Patient with MRSA Peritonitis and Bacteremia.. J Infect.

[39] Ammerlaan H, Seifert H, Harbarth S, Brun-Buisson C, Torres A (2009). Adequacy of antimicrobial treatment and outcome of Staphylococcus aureus bacteremia in 9 Western European countries.. Clin Infect Dis.

[40] Moellering RC (2009). What is inadequate antibacterial therapy?. Clin Infect Dis.

[41] Jia H (2008). Regulators scramble to tighten loopholes after heparin debacle.. Nat Biotechnol.

[42] United States Pharmacopeial Convention (2002). The Pharmacopoeia of the United States of America.

[43] Akers MJ (2002). Excipient-drug interactions in parenteral formulations.. J Pharm Sci.

[44] Crowley PJ (1999). Excipients as stabilizers.. Pharm Sci Technolo Today.

[45] Allen LV (2008). Dosage form design and development.. Clin Ther.

[46] Duenas-Laita A, Pineda F, Armentia A (2009). Hypersensitivity to generic drugs with soybean oil.. N Engl J Med.

[47] Napke E, Stevens DG (1984). Excipients and additives: hidden hazards in drug products and in product substitution.. Can Med Assoc J.

[48] Wang D, Notari RE (1994). Cefuroxime hydrolysis kinetics and stability predictions in aqueous solution.. J Pharm Sci.

[49] Recht MI, Douthwaite S, Puglisi JD (1999). Basis for prokaryotic specificity of action of aminoglycoside antibiotics.. EMBO J.

