# Supplementary material for: Determination of Therapeutic Equivalence of Generic Products of Gentamicin in the Neutropenic Mouse Thigh Infection Model
Source: PLoS One. 2010 May 20;5(5):e10744. doi: 10.1371/journal.pone.0010744 (PMC2873963; doi:10.1371/journal.pone.0010744)
Supplement: Table S1 — Detailed information of the products studied, their sources, and batches employed in vitro and in vivo. (0.05 MB PDF) [file pone.0010744.s001.pdf]

Supporting Table 1. Detailed information of the products studied, their sources, and batches employed in vitro and in vivo.

Gentamicin (GNT) Product*	Concentration labeled in ampoules for i.m. or i.v use	License number	Batch	No. of experiments using each batch		Manufacturer / Distributor
				In vitro	In vivo	
**GNT-S Plough (innovator)**	**160 mg / 2 ml**	**M-001143 R-2**	**CB0DPDC7<br>CB1DPDC7<br>CB1DPDC1<br>5DPDA010<br>7DPDA002**	**0<br>0<br>0<br>0<br>1**	**1<br>1<br>1<br>1<br>0**	**Schering-Plough SA, Bogota, Colombia / Idem**
	**120 mg / 1.5 ml**	**M-005324 R-1**	**CB1DPDC2<br>CB4DPDC06<br>6DPDA007**	**0<br>1<br>1**	**3<br>0<br>0**	
	**80 mg / 2 ml**	**M-001340-R2**	**CB2AMKB05<br>CB3AMKB04**	**1<br>1**	**0<br>1**	
GNT-Abbott	80 mg / 2 ml	06-6835-2/R3-1/91	75-024-DK	3	4	Abbott Laboratories, North Chicago, IL, USA / No distributor
GNT-Anglopharma	160 mg / 2 ml	M-003849	0101	0	2	Not described / Anglopharma SA, Bogota, Colombia
	80 mg / 2 ml	M-006642	35796<br>01-98	0<br>2	1<br>0	
GNT-AZ pharma*	120 mg / 2 ml	M-006660	0110059<br>009040*<br>303030	0<br>1<br>1	1<br>0<br>0	Vitrofarma SA, Bogota, Colombia / AZ-Pharma SA, Bogota, Colombia
GNT-Biochemie	80 mg / 2 ml	M-011772	07102321	3	2	Biochemie GmbH, Kundl, Austria / Novartis de Colombia SA, Bogota, Colombia
GNT-Biogenta	160 mg / 2 ml	M-007009-R1	0199<br>0299	2<br>0	0<br>2	Laboratorios Chalver de Colombia Ltda, Bogota Colombia / Idem
	80 mg / 2 ml	M-007036	0402	1	0	
GNT-Colmed	80 mg / 2 ml	M-006661	01005	3	1	Vitrofarma SA, Bogota, Colombia / Colmed Internacional by Procaps SA, Barranquilla, Colombia
GNT-Gencol	Not identified	Not identified	0100	2	1	Genericos de Colombia SA / Idem

GNT-Genfar	120 mg / 1.5 ml	M-013454-R1	041000<br>111200<br>051200<br>030703	1<br>0<br>1<br>1	0<br>1<br>0<br>1	Viteco SA, Bogota, Colombia / Laboratorios Genericos Farmaceuticos SA, Bogota, Colombia
GNT-Lab America	120 mg / 2 ml	M-002407	0100	2	2	Viteco SA, Bogota, Colombia / Laboratorios America SA, Medellin, Colombia
	80 mg / 2 ml	M-002414	0980303	1	0	Arbofarma SA, Colombia / Laboratorios America SA, Medellin, Colombia
GNT-Labinco	160 mg / 2 ml	M-007147	01013C	1	1	Laboratorios Ryan SC, Bogota, Colombia / Laboratorio Internacional de Colombia SA (Labinco), Bogota, Colombia
	120 mg / 3 ml	M-006138	01262MYO-1<br>01272 SPO	0<br>0	1<br>1	Viteco SA, Bogota, Colombia / Labinco
			01282FB1	2	0	Laboratorios Ryan de Colombia SC, Bogota, Colombia / Labinco
GNT-La Sante	160 mg / 2 ml	M-006698	0005<br>0310	0<br>1	1<br>0	Viteco SA, Bogota, Colombia / Laboratorios La Sante, Bogota, Colombia
	120 mg / 1.5 ml	M-006697	9901	2	0	
GNT-Memphis	160 mg / 2 ml	M-013859	16800900<br>02750200	0<br>2	1<br>0	Vitrofarma SA, Bogota, Colombia / Memphis products SA, Bogota, Colombia
	120 mg / 1.5 ml	M-014394	2208I10	1	0	
GNT-Merck	40 mg / 1 ml	M-002572 R-1	9838001	2	2	Merck Colombia SA, Bogota, Colombia under license of Merck KGaA, Darmstadt, Germany / Idem
GNT-MK	160 mg / 2 ml	M-011281 R-1	1C028	0	1	Tecnoquimica SA, Cali Colombia or Vitrofarma SA, Bogota, Colombia / Tecnoquimica SA, Cali Colombia
	60 mg / 1.5 ml	M-007357-R1	1E060	0	2	
	20 mg / 2 ml	M-007883-R1	3M053<br>3P066	2<br>1	0<br>0	
GNT-Ophalac	120 mg / 1.5 ml	M-007428	004013	1	0	Vitrofarma SA, Bogota, Colombia / Laboratorios Farmaceuticos Ophalac SA, Bogota, Colombia
	40 mg / 1 ml	M-007427	903005	2	1	

GNT-Pentacoop	160 mg / 2 ml	M-004112	10433<br>10023	0<br>0	1<br>1	Vitrofarma SA, Bogota, Colombia / Pentacoop SA, Bogota, Colombia
			32836<br>33544	2<br>1	0<br>0	Laboratorios Ryan de Colombia SC, Bogota, Colombia / Pentacoop SA, Bogota, Colombia
GNT-Rande	160 mg / 2 ml	M-012087	10200	2	2	Viteco SA, Bogota, Colombia / Laboratorios Rande Ltda, Medellin, Colombia
GNT-Recipe*	120 mg / 2 ml	M-006660	009040*<br>A120698	0<br>2	2<br>0	Vitrofarma SA, Bogota, Colombia / Linea Recipe® of Laboratorios Bussie SA, Colombia
	80 mg / 2 ml	M-006661	A030630<br>301094	0<br>1	1 (survival)<br>0	
GNT-Servipharm	40 mg / 1 ml	M-009220	07191751	2	2	Casar Laboratorios Ltda, Bogota, Colombia / Servipharm SA, subsidiary of CIBA-GEIGY Ltda, Basilea, Switzerland
GNT-Sigma	692 µg per mg	Not licensed for clinical use	10k1510	3	1	Sigma Chemical Co, St Louis, MO, USA / Idem

*This batch belongs to the same maker (Vitrofarma SA), share the same license number (M-006660), and are distributed by two vendors (AZ pharma and Recipe)
